# Primary School Education May Be Sufficient to Moderate a Memory-Hippocampal Relationship

**DOI:** 10.3389/fnagi.2018.00381

**Published:** 2018-11-20

**Authors:** Elisa de Paula França Resende, Howard J. Rosen, Kevin Chiang, Adam M. Staffaroni, Isabel Allen, Lea T. Grinberg, Karoline Carvalho Carmona, Henrique Cerqueira Guimarães, Viviane Amaral Carvalho, Maira Tonidandel Barbosa, Leonardo Cruz de Souza, Paulo Caramelli

**Affiliations:** ^1^Grupo de Pesquisa em Neurologia Cognitiva e do Comportamento, Departamento de Clínica Médica, Faculdade de Medicina da Universidade Federal de Minas Gerais, Belo Horizonte, Brazil; ^2^Memory and Aging Center, Department of Neurology, University of California, San Francisco, San Francisco, CA, United States

**Keywords:** education, cognitive reserve, aging, episodic memory, hippocampus, neuroimaging

## Abstract

According to the cognitive reserve theory, intellectual stimuli acquired during life can prevent against developing cognitive impairment. The underlying cognitive reserve mechanisms were underexplored in low-educated individuals. Because episodic memory impairment due to hippocampal dysfunction is a key feature of Alzheimer’s dementia (AD), we sought to look at a possible cognitive reserve mechanism by determining whether few years of education moderated the relationship between the hippocampal volumes and the episodic-memory scores. The sample was composed by 183 older adults, 40.1% male, with the median age of 78[76,82] years and the median years of education of 4[2,10] who had undergone an episodic-memory test and a 3-Tesla MRI scan to access the hippocampal volumes. Overall, 112 were cognitively healthy, 26 had cognitive impairment-no dementia (CIND) and 45 had dementia. We used multiple linear regression to assess whether the interaction between years of education and each hippocampal volume significantly predicted the episodic-memory scores’ variance, controlling for cognitive diagnosis and nuisance variables. The interaction term with the left hippocampus (ß = 0.2, *p* = 0.043, CI = 1.0, 1.4), but not with the right (ß = 0.1, *p* = 0.218, CI = 0.9, 1.2) significantly predicted the variation on memory scores. The mechanism by which the left hippocampus seems to play a more important role on memory processing in more educated individuals needs to be further investigated and might be associated with a better use of mnemonic strategies or higher hippocampal connectivity. Because the sample’s median years of education was four, which corresponds to primary school, we may infer that this level might be sufficient to contribute for building cognitive reserve.

## Introduction

Preventive interventions are becoming relevant as useful strategies to contain the rising dementia rates, given the constant failures of disease-modifying trials targeting Alzheimer’s dementia (AD). Preventive interventions should target specific mechanisms and prioritize modifying known risk factors like low education, that accounts for 19% of the AD’s crude population attributable risk (Norton et al., 2014). To develop strategies that will harness education as a preventative tool, we need to better understand the protective mechanisms of education. Cognitive reserve, a framework created to study those mechanisms (Stern, 2016), has the educational attainment as one of its main components. Some proposed underlying mechanisms are the development of better cognitive abilities (Nitrini et al., 2005; Opdebeeck et al., 2016; Stern, 2016) and a lesser cognitive impairment than expected due to cerebrovascular lesions (Farfel et al., 2013; Vaque-Alcazar et al., 2016), hippocampal atrophy (Murray et al., 2011) and amyloid pathology (Roe et al., 2008; Wirth et al., 2014) by more educated individuals. Other mechanisms are related to building brain reserve, supported by studies showing that the higher the education the larger the brain volumes, more connectivity between brain regions, the lower rate of hippocampal atrophy (Arenaza-Urquijo et al., 2013; Persson et al., 2016) and the more efficient the brain activation during memory tests (Springer et al., 2005; Bartrés-Faz et al., 2009).

However, most studies investigating the mechanisms of education as a cognitive reserve component have been conducted in high-income countries. Nevertheless, more than 60% of people with dementia live in low -and middle-income countries where 22–88% of the older adults are illiterate (UNESCO, 2016). Therefore, unveiling mechanisms of cognitive reserve in low-educated individuals can inform about the contribution of even few years for building reserve.

Considering that the hippocampi are key structures for episodic memory processing (Tulving, 2002; Sarazin et al., 2010) and that hippocampal dysfunction is a hallmark of AD (Dubois et al., 2010), we sought to look whether the years of formal education would moderate the relationship between hippocampal volumes and episodic-memory performance in a sample of older adults with a wide range of educational levels, from illiterate to college. We hypothesized that an increase in the number of years of education would lead to a stronger association between the hippocampal volumes and the episodic memory scores, suggesting a potential mechanism of cognitive reserve in the context of low educational attainment.

## Materials and Methods

### Participants

The participants (*n* = 183) came from two studies conducted in the Southeast region of Brazil, one from a tertiary memory clinic (*n* = 47) and other from the community (*n* = 136) (see Supplementary Data for details about the community-dwelling participants). There was not a minimum of years of education to be included in the study.

All participants were examined by a team of experienced board-certified neurologists, geriatricians, and one psychiatrist and they were divided into three cognitive groups: cognitively-healthy, cognitive impairment-no dementia (CIND) and dementia. The cognitively-healthy participants were functionally independent and scored within expected by age and education in the Brief Cognitive Battery (BCB) (Nitrini et al., 2004). The participants in the dementia group were functionally dependent and met the DSM-5 diagnostic criteria for dementia or major neurocognitive disorder (APA, 2013). The participants in the CIND group were functionally independent and performed lower than expected in the BCB test, therefore they all had objective cognitive impairment, most of them in the memory domain. Functional independence was defined as a score less than five in the Functional Activities Questionnaire (Pfeffer et al., 1982).

The Ethics Committee of the Federal University of Minas Gerais approved both studies and all participants, or their legally authorized representatives provided written informed consent, according to the principles of the Helsinki declaration.

### Cognitive Evaluation

All participants underwent the Mini-Mental State Examination (MMSE) (Folstein et al., 1975; Brucki et al., 2003) and the BCB. The BCB is a visual-verbal episodic memory test that does not suffer the influence of the educational level (Nitrini et al., 2004). We used the free delayed-recall scores as surrogates of episodic memory because of their ability to discriminate patients with AD from CIND and cognitively-healthy (Nitrini et al., 2004; Yassuda et al., 2017). Details about the BCB test are found in Supplementary Data.

### Neuroimaging

All participants’ scans were acquired in the same 3-Tesla Philips scanner. The 3D-T1 images were acquired in a sagittal plane (acquisition parameters in Supplementary Data) and were preprocessed in SPM-12, segmented into gray, white matter and cerebrospinal fluid, warped in the study-created template and normalized to the Montreal Neurologic Institute space. Bilateral brain regions important for memory processing and often affected by Alzheimer’s disease (Karow et al., 2010) namely the hippocampi, the inferior parietal and supramarginal gyri, the posterior cingulate, the precuneus, the middle temporal, the inferior temporal, the entorhinal, the parahippocampal, the fusiform, and the middle frontal cortical regions were extracted automatically using the Neuromorphometric atlas (Desikan et al., 2006).

White-matter lesions volumes were automatically segmented and quantified using the Lesion Segmentation Tool version 1.2.2 in SPM8 (Schmidt et al., 2012; Birdsill et al., 2014) based on the fluid-attenuated inversion recovery images (acquisition parameters in Supplementary Data).

### Statistical Analyses

Between-cognitive groups differences in demographics and clinical characteristics, that had non-normal distribution per Shapiro-Wilk, were compared using Kruskal-Wallis. Differences in the proportions of males were calculated with Chi-square. Differences in the hippocampal volumes adjusted for total intracranial volume (TIV), that had normal distribution per Shapiro-Wilk, were calculated using one-way ANOVA.

The associations between hippocampal volumes and episodic memory were calculated using univariate linear regression considering the episodic-memory scores as the outcome and each hippocampal volume (right and left) adjusting for TIV as separate predictors. Multiple linear regression models were adjusted for age, years of education, gender, the cognitive diagnosis, the MMSE, the BCB’s learning phase scores, the sum of the other brain regions extracted through the Neuromorphometric Atlas and the volume of white matter lesions.

The moderator effect of years of education was tested by adding the interaction term (years of education × each hippocampal volume) as separate predictors in the multiple linear regression models.

The level of significance was considered as *p* < 0.05 in two-tailed tests. The analyses were conducted in the software Rstudio, Version 1.1.414–©2009–2018.

## Results

Of the 183 participants, 40.1% (*n* = 75) were male, the median age was 78[76, 82] years old and the median years of education was 4[2, 10]. The years of education ranged from 0 to 20 years. Overall, 112 were cognitively healthy, 26 had CIND and 45 had dementia. The hippocampal volumes followed a continuum across the three cognitive groups: larger hippocampi in cognitively-healthy participants (Left hippocampus = 1.5 ± 0.1 cm^3^, Right hippocampus = 1.6 ± 0.1 cm^3^) followed by participants with CIND (Left hippocampus = 1.4 ± 0.1 cm^3^, Right hippocampus = 1.5 ± 0.1 cm^3^) followed by participants with dementia, who had the smaller hippocampi (Left hippocampus = 1.3 ± 0.1 cm^3^, Right hippocampus = 1.4 ± 0.1 cm^3^; Table 1).

**Table 1:** Demographics, clinical characteristics and hippocampal volumes by cognitive diagnosis.

Total *n* = 183	Cognitively healthy (*n* = 112)	CIND (*n* = 26)	Dementia (*n* = 45)	*p*-value
Sex male (%)	43 (38.4)	10 (38.5)	22 (48.9)	0.463
Age (years)^‡^§	78.0 [76.0, 81.2]	80.0 [78.2, 83.7]	77.0 [72.0, 82.0]	0.004
Education (years) ^†‡^§	4.0 [3.0, 8.2]	2.0 [0.0, 4.0]	8.0 [3.0, 14.0]	<0.001
MMSE^†‡^	26.5 [24.0, 28.0]	21.0 [17.0, 24.5]	24.0 [20.0, 26.0]	<0.001
BCB learning^†‡^§	23.0 [20.0, 25.0]	17.5 [15.0, 21.5]	17.0 [15.0, 20.0]	<0.001
BCB delayed recall^†‡^§	8.0 [7.0, 9.0]	6.0 [5.0, 7.0]	4.0 [3.0, 5.0]	<0.001
Right hippocampus^†‡^	1.6 (0.1)	1.5 (0.2)	1.4 (0.2)	<0.001
Left hippocampus^†‡^	1.5 (0.1)	1.4 (0.2)	1.3 (0.1)	<0.001
Volume of white matter lesions	5029.9 [1297.1, 11885.8]	6165.3 [2447.7, 20117.6]	1671.8 [277.9, 5489.1]	<0.001


*Age, education, the Mini-Mental State Examination (MMSE), the Brief Cognitive Battery (BCB) learning, delayed recall scores and the volume of white matter lesions are depicted in median [Interquartile intervals] and the between-group differences were calculated using Kruskal-Wallis. The left and right hippocampal volumes are depicted in mean (standard deviation) and the between-group differences were calculated using one-way ANOVA. The hippocampi volumes are corrected for the total intracranial volume and are shown in cm^3^. CIND, Cognitive impairment-no dementia. ^†^Cognitively healthy compared with CIND, ^‡^Cognitively healthy compared with Dementia, ^x^ CIND compared with dementia.*

There was a positive and significant association between each hippocampal volume and the episodic-memory scores. The left hippocampus predicted 27.3% of the scores’ variance (ß = 5.5, *p* < 0.001, CI = 61.6, 893.1) and the right hippocampus predicted 18.5% (ß = 4.1, *p* < 0.001, CI = 17.3, 239.0; Supplementary Table 1). In the adjusted models, in which 72.9% of the episodic-memory scores’ variance was explained by the model that included the left hippocampus and 72.5% were explained by the model that included the right hippocampus, the hippocampal volumes alone were no longer significant predictors (ß = 1.0, *p* = 0.111, CI = 0.8, 9.6 for the left and ß = -0.1, *p* = 0.827, CI = 0.3, 2.7 for the right; Supplementary Table 2).

**FIGURE 1:**
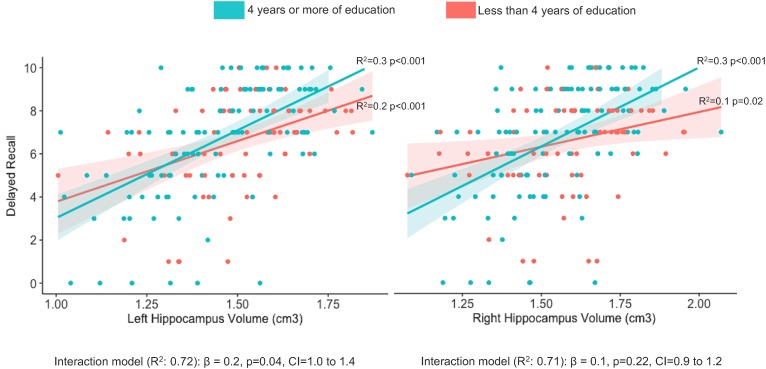
Visualization of the interaction between years of education and hippocampal volumes explaining the variation on episodic memory scores. The whole sample was split into those with less than four (red line) and those with four or more (green line) years of education to better visualize the moderator effect of education on the memory-hippocampal relationship. The fitted lines were calculated using the Spearman correlation test. In the multiple regression models controlling for age, years of education, gender, the cognitive diagnosis, the Mini-Mental State examination, the learning phase of the memory test, the sum of other brain regions important for memory processing and the volume of white matter lesions, the interaction term was significant for the left, but not for the right hippocampus.

In the models that included the interaction terms, the term that included the left hippocampus was a significant predictor of the episodic-memory scores’ variation (ß = 0.2, *p* = 0.043, CI = 1.0, 1.4), but the term that included the right hippocampus was not significant (ß = 0.1, *p* = 0.218, CI = 0.9, 1.2; Supplementary Table 3). To better visualize the moderator effect, we plotted in a graph (Figure 1) the hippocampal volumes in the X-axis and the episodic-memory scores in the Y-axis, adding one line that fitted the association between these two variables in participants who had less than four years of education and other line that fitted the association amongst participants who had 4 years or more. The fitted lines were calculated using the Spearman correlation test.

## Discussion

In a sample of older adults with a median of 4 years of formal education, which is the equivalent of primary school, we found that the longer the participants stayed in school, the stronger the association between the left hippocampal volume and the episodic-memory scores suggesting a potential cognitive reserve mechanism in this context. Because of this positive association, we speculate that the mechanism underlying reserve, in this case, is related to brain plasticity, denoting an increase in hippocampal involvement in memory processing in more educated individuals. Intellectual stimuli acquired in school might have allowed synapses and connections to develop, leading to higher hippocampal efficiency. This theory is supported by the fact that more educated individuals make better use of mnemonic strategies (Saczynski et al., 2007), which are thought to rely upon hippocampal functioning (Maguire et al., 2003) and to underlie more efficient memory processing (Bottiroli et al., 2008). Although these are tempting explanations, they carry a cause-effect association that our cross-sectional study was not designed to answer. A bidirectional relationship, as proposed in a previous study (Wenger and Lövdén, 2016), can also be driving our finding: In one direction, hippocampal efficiency predicts learning ability and lead to further achievement in intellectual life and in the other, cognitive stimulation result in a more efficient hippocampal functioning.

In our study, we found the moderator effect of education on the memory-hippocampal relationship to be significant only on the left side. One possible explanation is that the intellectual stimuli acquired in school is strongly related to writing and reading, cognitive abilities that are more left lateralized (Dehaene et al., 2010; Dehaene et al., 2015). Even mental arithmetic, an activity that is extensively trained in school and classically related to the right hippocampus (Supekar et al., 2013), has also been associated with increased connectivity in the left hippocampus (Klein et al., 2018). Therefore, it seems that the left hippocampus might be more stimulated in school-related activities. However, this conclusion will be possible to draw only in a study in which the school-related activities are better controlled and homogenous. For instance, training in a foreign language, that supposedly would be associated with left brain changes, has been associated with an increase in the right hippocampal volume in one study (Bellander et al., 2016) and an increase in both hippocampi in another (Martensson et al., 2012), reflecting the complexity of lateralization in learning language abilities.

Our finding can also be interpreted as counterintuitive because if an individual relies more on the hippocampus to process memory, once it is affected by a neurodegenerative disease, there is not much left as a reserve. In fact, contrary to our findings, a previous study showed that the higher the baseline intelligence measured at 20 years-old, the weaker the association between hippocampal volumes and episodic-memory scores in older adults (Vuoksimaa et al., 2013) suggesting that individuals with more cognitive reserve depend less on the hippocampus, which can be an advantage considering that this region is amongst the first to be affected by AD. However, while in our study low education was considered as less than 4 years, the above-mentioned study considered low education as less than 12 years. We propose that different mechanisms might explain the relationship between hippocampal volume and memory processing at different levels of education. At very low levels (e.g., less than 4 years), the hippocampal involvement in memory is independent of formal education and may be more related to inherited abilities (Van Petten, 2004). At intermediate levels, (e.g., 4–8 years), education and other cognitive stimuli can play a significant role in strengthening the hippocampus-memory relationship, which is supported by studies associating cognitive training with increasing in hippocampal volume (Draganski et al., 2006; Taubert et al., 2012). After high-school, there may be less of a relationship between additional improvement in memory abilities and hippocampal efficiency. At this point, either other brain regions may become more important in better memory processing (Wenger and Lövdén, 2016) or there may be a threshold of knowledge (e.g., mnemonic strategies) that improves hippocampal efficiency and little is added to the relationship with further education, so significant improvement cannot be observed. Indeed, a previous study showed that the relationship between cognitive performance and brain structure was more influenced by education in the less educated group (mean 7 years of education) than in the more educated (mean 13 years) (Mungas et al., 2009). Because of limitations in our sample size, we could not test this proposition that we hope to test it in future research.

Including participants with dementia and a high burden of white-matter lesions could have resulted in greater noise, reducing our ability to investigate the hippocampus-memory relationship, and may be considered a limitation of our study. However, we controlled for the cognitive diagnosis and the volume of white matter lesions in the regression model; therefore, our findings can be considered independent from these effects. Another limitation of our study is that we did not account for other possible cognitive reserve contributors such as occupational attainment and intellectual engagement in leisure activities. However, because our final models explained more than 70% of the variation on the episodic-memory scores, adding those other cognitive reserve components would be unlikely to significantly change our results. Unfortunately, an important limitation of our study was that we did not have information about the Intelligence Quotient (IQ) and the socioeconomic status of all the participants. People with lower educational levels probably had lower IQ and came from a lower socioeconomic background which is associated with worse nutrition and lack of access to adequate health care, which can also influence brain health. Indeed, it is difficult to disentangle the role of each component of cognitive reserve on shaping the relationship between a brain structure and a brain function. Although each aspect might play an independent and additive role, we believe that years of education is a reasonable proxy that reflect the overall consequences of a brain that was not adequately stimulated during childhood. Finally, another limitation of our study is that we did not perform a deep evaluation of the executive functioning of all our participants. It is known that executive functioning plays an important role on episodic memory processing through connections between the hippocampus and prefrontal regions (Buckner, 2004; Metzler-Baddeley et al., 2011). Therefore, in our study, we cannot disentangle the executive functioning component of the relationship between hippocampal size and episodic memory performance.

Our findings shed light on a potential cognitive reserve mechanism in the context of low education. Considering that the hippocampi are thought to preserve their plasticity even at later ages (Walhovd et al., 2016), developing preventive strategies focused on improving the educational level by few years may prevent the episodic memory impairment caused by AD in some cases, which is particularly important for underserved populations who have a low educational attainment and are facing increasing rates of dementia (Ferri and Jacob, 2017).

## Data Availability Statement

The raw data supporting the conclusions of this manuscript will be made available by the authors, without undue reservation, to any qualified researcher.

## Author Contributions

ER, HR, LCdS, and PC designed and performed the research, analyzed and interpreted the results, and wrote the paper. IA contributed to the statistical analysis. KCC contributed to the acquisition of the MRI scans and the participant’s evaluation. KC processed the MRI scans and calculated the brain volumes. HG and MB contributed to research design and participant’s evaluation. VC contributed to the neuropsychological evaluation of the participants. AS and LG critically reviewed the paper. All authors contributed to manuscript revision, read and approved the submitted version.

## Conflict of Interest Statement

The authors declare that the research was conducted in the absence of any commercial or financial relationships that could be construed as a potential conflict of interest.

## References

[B1] APA (2013). *Diagnostic and Statistical Manual of Mental Disorders.* 5th Edn, Arlington, VA: American Psychiatric Publishing.

[B2] Arenaza-Urquijo E. M. Landeau B. Mevel K. (2013). Relationships between years of education and gray matter volume, metabolism and functional connectivity in healthy elders. *Neuroimage* 83 450–457. 10.1016/j.neuroimage.2013.06.053 23796547

[B3] Bartrés-Faz D. Solé-Padullés C. Junqué C. (2009). Interactions of cognitive reserve with regional brain anatomy and brain function during a working memory task in healthy elders. *Biol. Psychol.* 80 256–259. 10.1016/j.biopsycho.2008.10.005 19022337

[B4] Bellander M. Berggren R. Martensson J. Brehmer Y. Wenger E. Li T. Q. (2016). Behavioral correlates of changes in hippocampal gray matter structure during acquisition of foreign vocabulary. *Neuroimage* 131 205–213. 10.1016/j.neuroimage.2015.10.020 26477659

[B5] Birdsill A. C. Koscik R. L. Jonaitis E. M. Johnson S. C. Okonkwo O. C. Hermann B. P. (2014). Regional white matter hyperintensities: aging, Alzheimer’s disease risk, and cognitive function. *Neurobiol. Aging* 35 769–776. 10.1016/j.neurobiolaging.2013.10.072 24199958 PMC3880609

[B6] Bottiroli S. Cavallini E. Vecchi T. (2008). Long-term effects of memory training in the elderly: a longitudinal study. *Arch. Gerontol. Geriatr.* 47 277–289. 10.1016/j.archger.2007.08.010 17936376

[B7] Brucki S. M. Nitrini R. Caramelli P. Bertolucci P. H. Okamoto I. H. (2003). Sugestões para o uso do mini-exame do estado mental no Brasil. *Arq. Neuropsiquiatr.* 61 777–781. 10.1590/S0004-282X2003000500014 14595482

[B8] Buckner R. L. (2004). Memory and executive function in aging and AD: multiple factors that cause decline and reserve factors that compensate. *Neuron* 44 195–208. 10.1016/j.neuron.2004.09.006 15450170

[B9] Dehaene S. Pegado F. Braga L. W. Ventura P. Nunes Filho G. Jobert A. (2010). How learning to read changes the cortical networks for vision and language. *Science* 330 1359–1364. 10.1126/science.1194140 21071632

[B10] Dehaene S. L. Cohen L. Morais J. Kolinsky R. (2015). Illiterate to literate: behavioural and cerebral changes induced by reading acquisition. *Nat. Rev. Neurosci.* 16 234–244. 10.1038/nrn3924 25783611

[B11] Desikan R. S. Ségonne F. Fischl B. Quinn B. T. Dickerson B. C. Blacker D. (2006). An automated labeling system for subdividing the human cerebral cortex on MRI scans into gyral based regions of interest. *Neuroimage* 31 968–980. 10.1016/j.neuroimage.2006.01.021 16530430

[B12] Draganski B. Gaser C. Kempermann G. Kuhn H. G. Winkler J. Büchel C. (2006). Temporal and spatial dynamics of brain structure changes during extensive learning. *J. Neurosci.* 26 6314–6317. 10.1523/JNEUROSCI.4628-05.2006 16763039 PMC6675198

[B13] Dubois B. Feldman H. H. Jacova C. Cummings J. L. Dekosky S. T. Barberger-Gateau P. (2010). Revising the definition of Alzheimer’s disease: a new lexicon. *Lancet Neurol.* 9 1118–1127. 10.1016/S1474-4422(10)70223-4 20934914

[B14] Farfel J. M. Nitrini R. Suemoto C. K. Grinberg L. T. Ferretti R. E. L. Leite R. E. P. (2013). Very low levels of education and cognitive reserve: a clinicopathologic study. *Neurology* 81 650–657. 10.1212/WNL.0b013e3182a08f1b 23873971 PMC3775692

[B15] Ferri C. P. Jacob K. S. (2017). Dementia in low-income and middle-income countries: different realities mandate tailored solutions. *PLoS Med.* 14:e1002271. 10.1371/journal.pmed.1002271 28350797 PMC5370095

[B16] Folstein M. F. Folstein S. E. McHugh P. R. (1975). Mini-mental state’. A practical method for grading the cognitive state of patients for the clinician. *J. Psychiatr. Res.* 12 189–198. 10.1016/0022-3956(75)90026-6 1202204

[B17] Karow D. S. McEvoy L. K. Fennema-Notestine C. Hagler D. J. Jennings R. G. Brewer J. B. (2010). Relative capability of MR imaging and FDG PET to depict changes associated with prodromal and early Alzheimer disease. *Radiology* 256 932–942. 10.1148/radiol.10091402 20720076 PMC2923729

[B18] Klein E. Willmes K. Bieck S. M. Bloechle J. Moeller K. (2018). White matter neuro-plasticity in mental arithmetic: changes in hippocampal connectivity following arithmetic drill training. *Cortex* 10.1016/j.cortex.2018.05.017 [Epub ahead of print]. 29961540

[B19] Maguire E. A. Valentine E. R. Wilding J. M. Kapur N. (2003). Routes to remembering: the brains behind superior memory. *Nat. Neurosci.* 6 90–95. 10.1038/nn988 12483214

[B20] Martensson J. Eriksson J. Bodammer N. C. Lindgren M. Johansson M. Nyberg L. (2012). Growth of language-related brain areas after foreign language learning. *Neuroimage* 63 240–244. 10.1016/j.neuroimage.2012.06.043 22750568

[B21] Metzler-Baddeley C. Jones D. K. Belaroussi B. Aggleton J. P. O’Sullivan M. J. (2011). Frontotemporal connections in episodic memory and aging: a diffusion MRI tractography study. *J. Neurosci.* 31 13236–13245. 10.1523/JNEUROSCI.2317-11.2011 21917806 PMC6623273

[B22] Mungas D. Reed B. R. Farias S. T. Decarli C. (2009). Age and education effects on relationships of cognitive test scores with brain structure in demographically diverse older persons. *Psychol. Aging* 24 116–128. 10.1037/a0013421 19290743 PMC2861868

[B23] Murray A. D. Staff R. T. McNeil C. J. Salarirad S. Ahearn T. S. Mustafa N. (2011). The balance between cognitive reserve and brain imaging biomarkers of cerebrovascular and Alzheimer’s diseases. *Brain* 134(Pt 12), 3687–3696. 10.1093/brain/awr259 22102649

[B24] Nitrini R. Caramelli P. Herrera E. Jr. Charchat-Fichman H. Porto C. S. (2005). Performance in Luria’s fist-edge-palm test according to educational level. *Cogn. Behav. Neurol.* 18 211–214. 16340394 10.1097/01.wnn.0000195292.48422.d5

[B25] Nitrini R. Caramelli P. Herrera Junior E. Porto C. S. Charchat-Fichman H. Carthery M. T. (2004). Performance of illiterate and literate nondemented elderly subjects in two tests of long-term memory. *J. Int. Neuropsychol. Soc.* 10 634–638. 10.1017/S1355617704104062 15327741

[B26] Norton S. Matthews F. E. Barnes D. E. Yaffe K. Brayne C. (2014). Potential for primary prevention of Alzheimer’s disease: an analysis of population-based data. *Lancet Neurol.* 13 788–794. 10.1016/S1474-4422(14)70136-X 25030513

[B27] Opdebeeck C. Martyr A. Clare L. (2016). Cognitive reserve and cognitive function in healthy older people: a meta-analysis. *Neuropsychol. Dev. Cogn. B Aging Neuropsychol. Cogn.* 23 40–60. 10.1080/13825585.2015.1041450 25929288

[B28] Persson N. Ghisletta P. Dahle C. L. Bender A. R. Yang Y. Yuan P. (2016). Regional brain shrinkage and change in cognitive performance over two years: the bidirectional influences of the brain and cognitive reserve factors. *Neuroimage* 126 15–26. 10.1016/j.neuroimage.2015.11.028 26584866 PMC4733615

[B29] Pfeffer R. I. Kurosaki T. T. Harrah C. H. Chance J. M. Filis S. (1982). Measurement of functional activities in older adults in the community. *J. Gerontol.* 37 323–329. 7069156 10.1093/geronj/37.3.323

[B30] Resende E. D. P. F. Chiang K. Allen I. Guimaraes H. C. Barbosa M. D. Carmona K. C. (2017). Education can strengthen the role of the left hippocampus in episodic memory performance. *Alzheimers Dement.* 13 761–762.28174069

[B31] Roe C. M. Mintun M. A. D’Angelo G. Xiong C. Grant E. A. Morris J. C. (2008). Alzheimer disease and cognitive reserve: variation of education effect with carbon 11-labeled pittsburgh compound B uptake. *Arch. Neurol.* 65 1467–1471. 10.1001/archneur.65.11.1467 19001165 PMC2752218

[B32] Saczynski J. S. Rebok G. W. Whitfield K. E. Plude D. L. (2007). Spontaneous production and use of mnemonic strategies in older adults. *Exp. Aging Res.* 33 273–294. 10.1080/03610730701318899 17497371

[B33] Sarazin M. Chauviré V. Gerardin E. Colliot O. Kinkingnéhun S. de Souza L. C. (2010). The amnestic syndrome of hippocampal type in Alzheimer’s disease: an MRI study. *J. Alzheimers Dis.* 22 285–294. 10.3233/JAD-2010-091150 20847406

[B34] Schmidt P. Gaser C. Arsic M. Buck D. Förschler A. Berthele A. (2012). An automated tool for detection of FLAIR-hyperintense white-matter lesions in multiple sclerosis. *Neuroimage* 59 3774–3783. 10.1016/j.neuroimage.2011.11.032 22119648

[B35] Springer M. V. McIntosh A. R. Winocur G. Grady C. L. (2005). The relation between brain activity during memory tasks and years of education in young and older adults. *Neuropsychology* 19 181–192. 10.1037/0894-4105.19.2.181 15769202

[B36] Stern Y. (2016). An approach to studying the neural correlates of reserve. *Brain Imaging Behav.* 11 410–416. 10.1007/s11682-016-9566-x 27450378 PMC5810375

[B37] Supekar K. Swigart A. G. Tenison C. Jolles D. D. Rosenberg-Lee M. Fuchs L. (2013). Neural predictors of individual differences in response to math tutoring in primary-grade school children. *Proc. Natl. Acad. Sci. U.S.A.* 110 8230–8235. 10.1073/pnas.1222154110 23630286 PMC3657798

[B38] Taubert M. Villringer A. Ragert P. (2012). Learning-related gray and white matter changes in humans: an update. *Neuroscientist* 18 320–325. 10.1177/1073858411419048 22013150

[B39] Tulving E. (2002). Episodic memory: from mind to brain. *Annu. Rev. Psychol.* 53 1–25. 10.1146/annurev.psych.53.100901.135114 11752477

[B40] UNESCO (2016). *50TH Anniversary Of International Literacy Day: Literacy Rates are on the Rise but Millions Remain Illiterate*. Paris: UNESCO.

[B41] Van Petten C. (2004). Relationship between hippocampal volume and memory ability in healthy individuals across the lifespan: review and meta-analysis. *Neuropsychologia* 42 1394–1413. 10.1016/j.neuropsychologia.2004.04.006 15193947

[B42] Vaque-Alcazar L. Sala-Llonch R. Valls-Pedret C. Vidal-Pineiro D. Fernandez-Cabello S. Bargallo N. (2016). Differential age-related gray and white matter impact mediates educational influence on elders’ cognition. *Brain Imaging Behav.* 11 318–332. 10.1007/s11682-016-9584-8 27535872

[B43] Vuoksimaa E. Panizzon M. S. Chen C. H. Eyler L. T. Fennema-Notestine C. Fiecas M. J. (2013). Cognitive reserve moderates the association between hippocampal volume and episodic memory in middle age. *Neuropsychologia* 51 1124–1131. 10.1016/j.neuropsychologia.2013.02.022 23499725 PMC3660613

[B44] Walhovd K. B. Krogsrud S. K. Amlien I. K. Bartsch H. A. Bjørnerud A. Due-Tønnessen P. (2016). Neurodevelopmental origins of lifespan changes in brain and cognition. *Proc. Natl. Acad. Sci. U.S.A.* 113 9357–9362. 10.1073/pnas.1524259113 27432992 PMC4995982

[B45] Wenger E. Lövdén M. (2016). The learning hippocampus: education and experience-dependent plasticity. *Mind Brain Educ.* 10 171–183. 10.1111/mbe.12112

[B46] Wirth M. Villeneuve S. La Joie R. Marks S. M. Jagust W. J. (2014). Gene-environment interactions: lifetime cognitive activity, APOE genotype, and β-amyloid burden. *J. Neurosci.* 34 8612–8617. 10.1523/JNEUROSCI.4612-13.2014 24948815 PMC4061397

[B47] Yassuda M. S. da Silva H. S. Lima-Silva T. B. Cachioni M. Falcão D. V. D. S. Lopes A. (2017). Normative data for the brief cognitive screening battery stratified by age and education. *Dement. Neuropsychol.* 11 48–53. 10.1590/1980-57642016dn11-010008 29213493 PMC5619214

