# Supplementary material for: Primary School Education May Be Sufficient to Moderate a Memory-Hippocampal Relationship
Source: Front Aging Neurosci. 2018 Nov 20;10:381. doi: 10.3389/fnagi.2018.00381 (PMC6255790; doi:10.3389/fnagi.2018.00381)
Supplement: Supplementary file 1 [file Data_Sheet_1.docx]

Supplementary Material

**Primary school education may be sufficient to moderate a memory-hippocampal relationship**

**Elisa de Paula Franca Resende, Howard J. Rosen, Kevin Chiang, Adam M. Staffaroni, Isabel Allen, Lea T. Grinberg, Karoline Carvalho Carmona, Henrique Cerqueira Guimarães, Viviane Amaral Carvalho, Maira Tonidandel Barbosa, Leonardo Cruz de Souza, Paulo Caramelli***

*** Correspondence:** Dr. Paulo Caramelli, caramelli@ufmg.br

# The participants from the community-based study

The participants from the community-based study was a subsample of the Pietà study (Caramelli et al. 2011) which had a total of 639 participants, which corresponded to 51.1% of a city’s population aged 75+ years-old. The subsample was composed by the participants who had MRI scans (n=189) excluded the ones for whom the interval MRI-cognitive test was more than one year (n=53).

# The Brief Cognitive Battery test (BCB)

# The episodic memory test consists of presenting to the participant a paper with 10 simple drawings that have to be identified and named. Immediately after naming the drawings, the participant is asked to recall them (incidental encoding). The drawings are shown again and the participant is instructed to memorize the drawings for 30 seconds and recall them (immediate memory). This latter procedure is repeated once (learning). The BCB’s learning phase is composed by the sum of the incidental encoding, immediate memory and learning scores. Five minutes after recalling the drawings for the last time, after doing the clock drawing and the animals-fluency/minute tests, the participant is asked to freely recall the drawings (episodic memory). Finally, the participant is confronted with another paper containing the 10 previously presented drawings along with 10 distractors and is asked to recognize the initial ones (recognition). The recognition score is calculated by subtracting the number of drawings correctly recognized by the number of false recognitions.

# Neuroimaging acquisition parameters

**3D-T1** acquisition parameters: sagittal plane acquisition with spin-echo echoplanar sequences (TR/TE=16/4ms, matrix=240x240, slice thickness=1.0mm, 1.0mm gap between slices) and flip angle of 8^o^.

**Fluid-attenuated inversion recovery** images acquisition parameters: axial plane acquisition with a single-shot, spin-echo echoplanar sequences in the axial plane (TR/TE=10000/140ms, Inversion time:2800ms, Echo train length=27mm, FO=240mm, matrix=352x212 (reconstructed 512x512), slice thickness=5.0 mm, 6.0mm gap between slices).

**Supplementary tables:**

**Table 1: Univariate regression models showing the association between the hippocampal volumes and the scores on episodic memory tests**

|  | Univariate Regression models | | | | | | | | |
| --- | --- | --- | --- | --- | --- | --- | --- | --- | --- |
|  | **Beta coefficient** | **Confidence Interval** | **p value** |  |  | **Beta coefficient** | **Confidence Interval** | **p value** |  |
| Left hippocampus | 5.5 | 61.6;893.1 | <0.001 |  | **Right hippocampus** | 4.1 | 17.3;239.0 | <0.001 |  |
| Total intracranial  volume | -0.0 | 0.9;0.9 | <0.001 |  | Total intracranial volume | -0.0 | 0.9;0.9 | <0.001 |  |

**Table 2: Multivariate regression models showing the association between the hippocampal volumes and the scores on episodic memory tests**

| Multivariate regression models | | | | | | | | | |
| --- | --- | --- | --- | --- | --- | --- | --- | --- | --- |
|  | **Beta coefficient** | **Confidence Interval** | **p value** |  |  | **Beta coefficient** | **Confidence Interval** | **p value** |  |
| Left hippocampus | 1.0 | 0.8;9.6 | 0.111 |  | **Right hippocampus** | -0.1 | 0.3;2.7 | 0.827 |  |
| Education | -0.0 | 0.9;1.0 | 0.367 |  | Education | -0.0 | 0.9;1.0 | 0.213 |  |
| Age | -0.0 | 0.9;1.0 | 0.547 |  | Age | -0.0 | 0.9;1.0 | 0.360 |  |
| Sex (Male) | -0.1 | 0.6;1.6 | 0.836 |  | Sex (Male) | -0.1 | 0.5;1.5 | 0.694 |  |
| Diagnosis  (Cognitively healthy) | -0.1 | 0.5;1.8 | 0.790 |  | Diagnosis  (Cognitively healthy) | 0.1 | 0.5;2.0 | 0.904 |  |
| Diagnosis  (Dementia) | -1.3 | 0.1;0.5 | <0.001 |  | Diagnosis  (Dementia) | -1.4 | 0.1;0.5 | <0.001 |  |
| BCB learning | 0.3 | 1.3;1.4 | <0.001 |  | BCB learning | 0.3 | 1.3;1.4 | <0.001 |  |
| MMSE | 0.1 | 1.0;1.2 | 0.036 |  | MMSE | 0.1 | 1.0;1.2 | 0.038 |  |
| Total intracranial  volume | 0.0 | 0.9;1.0 | 0.435 |  | Total intracranial  volume | 0.0 | 0.9;1.0 | 0.233 |  |
| Volume of white matter lesions | -0.0 | 0.9;1.0 | 0.972 |  | Volume of white matter lesions | -0.0 | 0.9;1.0 | 0.920 |  |
| Sum of other cortical regions | -0.0 | 0.9, 1.0 | 0.295 |  | Sum of other cortical regions | -0.0 | 0.9;1.0 | 0.765 |  |

**Table 3: Model with the interaction term (hippocampal volume x years of education) suggesting the moderator effect of years of education on the association between the hippocampal volumes and the scores on the episodic memory tests**

| Multivariate regression models | | | | | | | | |  |
| --- | --- | --- | --- | --- | --- | --- | --- | --- | --- |
|  | **Beta coefficient** | **Confidence Interval** | **p value** |  |  | **Beta coefficient** | **Confidence Interval** | **p value** | |
| Left hippocampus | 0.1 | 0.2;4.9 | 0.920 |  | Right hippocampus | -0.1 | 0.1;2.1 | 0.405 | |
| Interaction:  Left hippocampus x education | 0.2 | 1.0;1.4 | 0.043 |  | Interaction:  Right hippocampus x education | 0.1 | 0.9;1.3 | 0.218 | |
| Education | -0.0 | 0.5;0.9 | 0.031 |  | Education | -0.0 | 0.5;1.1 | 0.152 | |
| Age | -0.0 | 0.9;1.0 | 0.634 |  | Age | -0.0 | 0.9;1.0 | 0.381 | |
| Sex (Male) | -0.1 | 0.6;1.6 | 0.850 |  | Sex (Male) | -0.1 | 0.5;1.5 | 0.717 | |
| Diagnosis  (Cognitively healthy) | -0.0 | 0.5;1.9 | 0.962 |  | Diagnosis  (Cognitively healthy) | 0.1 | 0.5;2.2 | 0.780 | |
| Diagnosis  (Dementia) | -1.1 | 0.2;0.7 | 0.005 |  | Diagnosis  (Dementia) | -1.3 | 0.1;0.6 | 0.001 | |
| BCB learning | 0.3 | 1.3;1.4 | <0.001 |  | BCB learning | 0.3 | 1.3;1.4 | <0.001 | |
| MMSE | 0.1 | 1.0;1.2 | 0.020 |  | MMSE | 0.1 | 1.0;1.2 | 0.037 | |
| Total intracranial  volume | 0.0 | 0.9;1.0 | 0.359 |  | Total intracranial  volume | 0.0 | 0.9;1.0 | 0.209 | |
| Volume of white matter lesions | -0.0 | 0.9;1.0 | 0.872 |  | Volume of white matter lesions | -0,0 | 0.9;1.0 | 0.795 | |
| Sum of other cortical regions | -0.0 | 0.9, 1.0 | 0.200 |  | Sum of other cortical regions | -0.0 | 0.9;1.0 | 0.613 | |

**References**

Caramelli, Paulo, Maira Tonidandel Barbosa, Emilia Sakurai, Etelvina Lucas dos Santos, Rogerio Gomes Beato, Joao Carlos Barbosa Machado, Henrique Cerqueira Guimarães, and Antonio Lucio Teixeira. 2011. "The Pietà study. Epidemiological investigation on successful brain aging in Caeté (MG), Brazil. Methods and baseline cohort characteristics." *Arq Neuropsiquiatr.* 69 (4):579-584. doi: 10.1590/S0004-282X2011000500002.
